# Development of Oral Dissolvable Films of Diclofenac Sodium for Osteoarthritis Using* Albizia *and* Khaya *Gums as Hydrophilic Film Formers

**DOI:** 10.1155/2016/6459280

**Published:** 2016-05-30

**Authors:** Martina Aduenimaa Bonsu, Kwabena Ofori-Kwakye, Samuel Lugrie Kipo, Mariam El Boakye-Gyasi, Mary-Ann Fosu

**Affiliations:** Department of Pharmaceutics, Faculty of Pharmacy and Pharmaceutical Sciences, College of Health Sciences, Kwame Nkrumah University of Science and Technology (KNUST), Kumasi, Ghana

## Abstract

Oral dissolvable films (ODFs) of diclofenac sodium intended for osteoarthritis were prepared using* Albizia* and* Khaya* gums as hydrophilic film formers. The physicochemical properties of the gums were characterized and the gums were used to prepare diclofenac sodium ODFs (~50 mg/4 cm^2^ film) by solvent casting. The two gums showed satisfactory film forming properties. The physicomechanical properties, drug-excipient compatibility, and* in vitro* drug release of the films in phosphate buffer pH 6.8 were studied.* Khaya* gum had higher extraction yield, moisture content, insoluble matter and true density while* Albizia* gum showed greater swelling capacity, solubility, and minerals content. The ODFs were thin, soft, and flexible with smooth glossy surfaces and possessed satisfactory physicomechanical properties. FTIR studies showed that no interaction occurred between the drug and the gums. The ODFs disintegrated in <45 s achieved >75% drug release within 7 min with dissolution efficiencies of ~83–96%. Drug releases from F2, F3, F4, F5, and F6 were similar to F1 (*p* > 0.05; *f*1 < 15 and *f*2 ≥ 50) while F7 differed markedly from F1 (*p* < 0.001; *f*1 > 15 and *f*2 < 50). Drug release followed the Higuchi kinetic model which is indicative of Fickian drug diffusion.

## 1. Introduction 

Fast dissolving drug delivery systems like oral dissolvable films (ODFs) and fast disintegrating tablets (FDTs) were introduced in the late 1970s as alternative dosage forms for paediatric and geriatric patients who have difficulties in swallowing conventional oral solid dosage forms such as tablets and capsules [1]. ODFs are presented as thin flexible solid dosage forms and come in different sizes and shapes [2]. ODFs are administered by placing the dosage form on the tongue where they hydrate and break up releasing the active agent into the buccal cavity for oromucosal absorption or swallowing [3]. ODFs emerged from the confectionery and oral care department in the form of breath strips and soon grew into a contemporary and widely accepted dosage form by end users for delivering vitamins and personal care products [1]. They are composed basically of the active pharmaceutical ingredient (API), hydrophilic polymers, plasticizers, sweeteners, flavours, colours, surfactants, saliva stimulating agents, and so forth [4, 5].

ODFs are easy to transport and offer fast, safe, and accurate dosing without the need for water or any measuring device. Also, dysphagic, schizophrenic, and dementia patients are able to use ODFs with little or no difficulty [6]. In addition, they exhibit enhanced bioavailability as they bypass the hepatic first pass metabolism [7]. The main disadvantages of ODFs are the difficulty in attaining uniform dosage and achieving high drug loadings in the films. Also, there is a challenge with packaging of the films which may demand the use of special packaging machines [8]. Many drugs have been formulated as ODFs; these include NSAIDs, antihistamines, antiulcer drugs, nicotine replacements, antiemetics, antipsychotics, anti-Alzheimer drugs, antiepileptic drugs, and drugs for sleeping disorders [9–11].

Gums are pathological compounds produced by certain plants as a result of mechanical injury or due to undesirable climatic conditions, such as drought [12]. Examples include* Acacia*, tragacanth,* Albizia*,* Khaya*, xanthan, and guar gums. Gums find wide applications in the pharmaceutical industry where they are used as viscosity enhancers in some suspensions, as emulsifying agents in stabilizing emulsions, as binding agents in tablets and capsules, and so forth [13]. Gums have also shown great potential as gelling agents, film forming agents in ODF technology, coating agents in tablet cores intended for colonic drug delivery [14, 15], and also vehicles in the fabrication of modified release dosage forms [16].

*Albizia *gum is obtained as natural exudates from the incised trunk of* Albizia zygia* (DC.) J. F. Macbr., family Leguminosae. The plant is widely distributed in tropical Africa, and it is found in Senegal through to Kenya, northern Angola, and Tanzania [17].* Khaya *gum is obtained from the incised trunk of* Khaya grandifoliola* CDC, family Meliaceae. The tree is found in Benin, Democratic Republic of Congo, Côte D'Ivoire, Ghana, Togo, Nigeria, Sudan, Guinea, and Uganda [18].* Albizia *and* Khaya *gums have been evaluated as binding agents [13], thickening agents [16], nonfunctional film coatings [19, 20], and compression coatings for colonic drug delivery [14].

Osteoarthritis is the third leading diagnosis in the aged population [21] and causes substantial amount of pain leading to impairment and reduction in the quality of life of patients above 65 years [22]. NSAIDs such as diclofenac, aceclofenac, ibuprofen, and celecoxib are widely used to alleviate the pain of osteoarthritis. These drugs relieve inflammatory pain and slow down the inflammatory process and its associated tenderness and joint constraints. One of the challenges facing geriatric patients is the difficulty in swallowing conventional tablets which affects medication compliance and hinders good therapeutic outcomes. The formulation of active pharmaceutical ingredients (APIs) as ODF formats can help to resolve medication noncompliance in the elderly [1].

This study was aimed at developing oral dissolvable films of diclofenac sodium using* Albizia * and* Khaya *gums as natural hydrophilic film forming polymers. The developed ODFs are intended for the management of osteoarthritis in the elderly.

## 2. Materials and Methods 

### 2.1. Materials

Diclofenac sodium was received as a gift from Trade Winds Chemists (Kumasi, Ghana). Hydroxypropyl methylcellulose (HPMC) E15 (viscosity of 2% solution at 15 cPs) was obtained as a gift from UK Chemicals Ltd. (Kumasi, Ghana). Aspartame was a gift from Aspee Pharmaceuticals Ltd. (Kumasi, Ghana). Pineapple flavour and titanium dioxide were supplied by Kinapharma Ltd. (Accra, Ghana). Glycerol, Tween 80, and citric acid were obtained from the chemical stores of the Departments of Pharmaceutics and Pharmaceutical Chemistry, KNUST (Kumasi, Ghana). All other reagents used were of analytical grade. Crude* Albizia *and* Khaya *gums were obtained from forest areas around Kwahu in the Eastern Region of Ghana, as natural exudates from the incised trunks of the trees* Albizia zygia* (DC.) J. F. Macbr., family Leguminosae, and* Khaya grandifoliola* CDC, family Meliaceae, respectively. The gums were authenticated, collected, and supplied by a technician at the Department of Herbal Medicine, Faculty of Pharmacy and Pharmaceutical Sciences, KNUST, Ghana.

### 2.2. Methods

#### 2.2.1. Purification of Gums

Crude* Albizia *and* Khaya *gums were freed from extraneous materials and purified using methods described elsewhere [23, 24], with minor modifications. Gums were oven-dried at 50°C for 48 h and sorted into light and dark coloured grades. Eight hundred grams of the light coloured grades was separately milled and hydrated with 2 L double strength chloroform for 48 h, with occasional stirring. The gum mucilage substances were filtered with calico and the filtrates were precipitated with three times their volumes of ethanol, refiltered, and washed with diethyl ether. The resultant gums were oven-dried at 40°C for 24 h, milled, and screened through 250 *μ*m sieves, packed in airtight plastic pouches, and stored in a desiccator ready for use.

#### 2.2.2. Moisture Content and Insoluble Matter

The moisture content and insoluble matter in the gums were determined using British Pharmacopoeia methods [25].

#### 2.2.3. pH and Ash Values Determination

One gram of the gum was dispersed in sufficient distilled water, with occasional agitation, to form 1% w/v gum mucilage. The pH of the resultant mucilage was determined with a calibrated Eutech pH meter (pH 510, pH/mV/°C meter, Singapore) at 25°C. The total ash, acid insoluble ash, and water soluble ash of the gums were determined using official methods [25].

#### 2.2.4. True Density and Solubility Determination

The true density of the gums was determined by liquid displacement method at 25°C [26] and calculated as the weight of gum divided by the weight of the liquid it displaces. Chloroform was used as the displacement liquid as the gums are practically insoluble in the solvent. The solubility of the purified gums was determined in cold and warm water, acetone, chloroform, and ethanol (96%). One gram of the gum was added to 50 mL of the solvent and left overnight at 25°C. Twenty-five-milliliter portions of the clear supernatant were placed in preweighed glass petri dishes and evaporated to dryness over a water bath. The mass of the residues was weighed with an analytical balance (Adam Equipment, UK) and expressed as the percentage solubility of the gum in the solvent.

#### 2.2.5. Swelling Index

One gram of the gum powder was weighed into a 10 mL measuring cylinder. The initial volume of the gum was noted after which distilled water was added to the 10 mL mark. The cylinder was stoppered, mixed lightly, and allowed to stand for 24 h and the final volume occupied by the gum sediment was noted. The swelling index was calculated as follows: Swelling  Index = (*X*_*t*_ − *X*_*o*_/*X*_*o*_) × 100, where *X*_*t*_ is volume of gum after 24 h and *X*_*o*_ = initial volume of gum.

#### 2.2.6. Charring Temperature

The charring temperature was determined by the open capillary method using the Stuart melting point apparatus (Bibby Scientific Ltd., UK). An open capillary tube was sealed using a Bunsen burner. The tube was packed by pressing the open end gently into a sample of the dry powder gum. The gum was transferred from the open end to the bottom of the tube by gently tapping the bottom on the bench. The sample tube was then inserted into the melting point apparatus and the temperature at which the gum sample changed colour was determined.

#### 2.2.7. Viscosity Measurements

The viscosity of 5, 10, 20, 30, and 40% gum mucilage was measured with a Brookfield viscometer (Brookfield Engineering Laboratories, Inc., Middleboro, USA) at 25°C and shear rate of 30 rpm. The effect of temperature on the viscosity of 40% gum mucilage was also studied at 25, 50, 65, 75, and 85°C with the Brookfield viscometer at a shear rate of 30 rpm.

#### 2.2.8. Determination of Mineral and Toxic Ion Content

The mineral and toxic ion contents of the gums, namely, iron (Fe), copper (Cu), zinc (Zn), manganese (Mn), cadmium (Cd), lead (Pb), mercury (Hg), and arsenic (As), were determined with an atomic absorption spectrophotometer (AAS) (Buck Scientific Model 210V GP). The gums were subjected to dry ash digestion and the clear supernatant digest after centrifugation was used for the analysis. The file for the AAS analysis and hollow cathode lamps were set as follows: Fe at 248.3 nm, Cu at 324.8 nm, Zn at 213.9 nm, Mn at 279.5 nm, Cd at 228.9 nm, Pb at 283.3 nm, Hg at 253.7 nm, and Ar at 193.7 nm. A calibration curve was plotted for each of the elements to be analyzed from the stock standards. The stock standard followed by the sample solutions was analyzed for the elements. The determinations were done in triplicate for each supernatant digest. The contents of potassium and sodium in the gums were determined by flame photometry (Jenway flame photometer PFP7) at wavelengths of 766 nm and 589 nm, respectively. The content of phosphorus was evaluated using the phosphovanadomolybdate method [27] for colour development and absorbance was determined spectrophotometrically (Jenway 6051 colorimeter) at 430 nm. The nitrogen and organic carbon contents of the gums were determined by the Kjeldahl method and the modified Walkley-Black wet oxidation method, respectively [28].

### 2.3. Preparation of Diclofenac Sodium ODFs

Diclofenac sodium ODFs were prepared using the solvent casting method [7, 29], with minor modifications.  Table 1 shows the composition of the seven ODF formulations prepared. The polymers were immersed in half the volume of distilled water overnight to obtain a homogeneous dispersion. The specified amount of glycerol was added to the aqueous dispersion and mixed until being homogeneous. The aqueous solution was allowed to stand for 1 h to take out all entrapped air bubbles. Another aqueous solution was made by dissolving the specified amounts of diclofenac sodium, aspartame, titanium dioxide, flavour, Tween 80, and citric acid in the remainder of the distilled water. Both aqueous solutions were put together, stirred, and sonicated for 30 min. Twenty-milliliter portions of the resultant aqueous dispersion were cast onto glass petri dishes of diameter 90 mm and oven-dried at 60°C for 24. The dried films were meticulously taken out of the petri dishes, inspected for any imperfections, and cut into 2 cm × 2 cm sizes with a scalpel. The cut films were packaged singly in aluminium foils and kept in a desiccator pending assessment.

### 2.4. Evaluation of Films

#### 2.4.1. Appearance and Uniformity of Thickness

All prepared films were checked for their appearances, whether uniform or not, and for the presence or absence of air bubbles, and so forth. The thickness of five randomly selected 2 cm × 2 cm films from each ODF formulation was determined using a digital caliper (Powerfix, Milomex Ltd., UK). The measurements were taken along various planes of the films and the mean and standard deviation were calculated [30].

#### 2.4.2. Determination of Mean Weight and pH

The individual weights of ten randomly selected 2 cm × 2 cm films were determined using an analytical balance (Adam Equipment, UK). The average weight and standard deviations were calculated. The pH of the films was determined by dissolving a 2 cm × 2 cm film in 10 mL of distilled water. The pH of the resulting solution was measured using a standardized Eutech pH meter (pH 510, pH/mV/°C meter, Singapore). The mean of five determinations of each film was calculated.

#### 2.4.3. Drug-Excipient Compatibility Studies Using FTIR Spectroscopy

A Bruker FTIR spectrophotometer (Alpha-Platinum ATR, Jos Hansen & Soehne GmbH, Hamburg, Germany) run by the Opus software (Version 7.2 Build 7.2. 139. 1294) was set to baseline to format previous entries that may interfere with the determination. About 0.1 g of diclofenac sodium was loaded onto the stage directly on top of the platinum. The force gauge was pulled closer to the sample to compress the sample. When the setup was ready, the Opus software generated the spectrum of the loaded sample on the monitor of a computer. The FTIR spectra of both* Albizia *(F4) and* Khaya *(F6) films (each containing diclofenac sodium as API) were also determined to assess possible interaction between diclofenac sodium and the excipients in the formulation [3]. The spectra of all three samples were superimposed using the Opus software to make comparison of the spectra easier. The spectra were compared on the basis of whether or not the principal bands present in the pure diclofenac sodium were still present in the formulated diclofenac sodium ODFs.

#### 2.4.4. Disintegration Time of Films

The petri dish method was employed in the determination of the disintegration time of the ODFs. Five randomly selected 2 cm × 2 cm films were placed into 25 mL distilled water in a petri dish at 37 ± 0.5°C. The petri dish and its contents were swirled gently every 10 seconds till the film started to break up. The time taken by the film to break up completely was recorded as the disintegration time [4].

#### 2.4.5. Assay of ODFs

Five randomly selected 2 cm × 2 cm diclofenac sodium ODFs each containing ~50 mg diclofenac sodium were placed in separate conical flasks containing 70 mL of 0.1 M sodium hydroxide. The flasks were shaken for 15 min using a shaker. Sufficient quantities of 0.1 M NaOH were added to produce 100 mL in each flask. The resultant solutions were filtered and 2 mL of each filtrate was diluted to 100 mL with 0.1 M NaOH. The drug concentrations were evaluated spectrophotometrically (T90 UV/VIS spectrometer, PG Instruments Ltd., UK) at a wavelength of 276 nm using the regression data of the calibration curve (*y* = 336.9*x* − 0.0715, *R* ^2^ = 0.9971) [31].

#### 2.4.6. Evaluation of Mechanical Properties

The mechanical properties of 2 cm × 2 cm ODFs were evaluated using a Brookfield Texture Analyzer CT3-100 with TexturePro CT Software (Brookfield Engineering Lab. Inc., Middleboro, MA, USA), fitted with TA-DE and TA-DGA probes and equipped with a 10 kg load cell. The required test parameters were entered into the Texture Loader Software and the appropriate mode was chosen. In the measurement of tensile strength and elastic modulus, a randomly selected film was held between two clamps of probe TA-DGA using low pressure clips, allowing a distance of 3 cm between the sample surface and the base of the probe. For the elongation at break, a film was clamped between the accessory fixtures of probe TA-DE. During measurement, the film was pulled at a rate of 2 mm/s. The force at break and elongation were shown on the CT3 display when the film broke. With an attached computer and TexturePro CT software, the mechanical parameters, namely, tensile strength, elastic modulus, and percentage elongation, were obtained. The experiment was carried out in triplicate for each ODF formulation and the average and standard deviations were calculated [32]. The folding endurance of 2 cm × 2 cm films was determined by folding the film of uniform cross-sectional area and thickness in the same place until it broke. The number of times the film was folded in the same plane (before breaking) was recorded as the folding endurance of that film. Ten randomly sampled 2 cm × 2 cm films of uniform thickness per formulation were used and the average of the determinations was recorded as the folding endurance.

#### 2.4.7. *In Vitro* Release Studies

*In vitro* drug release studies on the ODFs were conducted with an Erweka dissolution apparatus (Type DT6, GmbH, Heusenstamm, Germany). Six 2 cm × 2 cm films of each formulation were evaluated. A film was placed with the help of forceps into a vessel containing 300 mL phosphate buffer pH 6.8, maintained at 37 ± 0.5°C with a stirring rate of 100 rpm. At 0, 7, 14, 21, and 28 min, 10 mL aliquots of the dissolution medium were withdrawn and replaced with an equal volume of fresh medium maintained at 37 ± 0.5°C. The withdrawn samples were filtered using Whatman filter paper (number. 5) and 5 mL of the filtrates was diluted ten times with phosphate buffer pH 6.8. The absorbance of the diluted filtrates was determined with a UV-visible spectrophotometer (T90 UV/VIS spectrometer, PG Instruments Ltd., UK) at a wavelength of 276 nm. Using the regression data obtained from the calibration curve (*y* = 333.97*x* − 0.0686, *R* ^2^ = 0.9981), the amount of diclofenac sodium in the samples was calculated. The percentage of diclofenac sodium released with reference to the expected content in each film was then calculated. A graph of cumulative mean percentage content of drug dissolved against the respective time points was plotted for each formulation to obtain the release profiles using GraphPad Prism Version 5.00 (GraphPad Software, Inc., USA).

### 2.5. Data Analysis

#### 2.5.1. Model Independent Approaches

Dissolution efficiency (DE), difference (*f*1), and similarity (*f*2) factors and one-way analysis of variance (ANOVA) were used to analyze the drug release data.

The dissolution efficiency was calculated using the equation

(1) $$\begin{array}{c} DE=\left\{\;\frac{\left(0\int tY\;dt\right)}{Y100}\cdot \left(\;t_{2}-t_{1}\;\right)\;\right\}\times 100, \end{array}$$

where (0∫*tY* *dt*) is area under the dissolution curve (AUC); *Y* is the percentage dissolved at *t*_2_; *t*_2_ is time for all active ingredient to dissolve; *t*_1_ is time at which first sample was withdrawn.

The difference and similarity factors were calculated using the following equations:

(2) f1=St=ln⁡Rt−TtSt=ln⁡Rt×100f2=50×log⁡1+1nSt=ln⁡Rt−Tt2−0.5×100,

where *n* is time points; *R*_*t*_ is cumulative percentage dissolved at time *t* for the reference; and *T*_*t*_ is cumulative percentage dissolved at time *t* for the test.

The drug release data were also subjected to one-way ANOVA followed by Dunnett's multiple comparison test using GraphPad Prism Version 5.00 (GraphPad Software, Inc., USA). Paired samples with *P* < 0.05 were considered to be significantly different.

#### 2.5.2. Model Dependent Approach

The drug release data were fitted into the zero-order, first-order, Higuchi, and Hixson-Crowell kinetic models to determine the release mechanism [33]. The model that produced good linearity indicated by a high *R* ^2^ value was considered the best model to describe the release kinetics of the formulated films.

## 3. Results and Discussion

*Albizia *and* Khaya *gums were collected, purified, and employed in the preparation of the diclofenac sodium ODFs.

### 3.1. Physicochemical Properties of Gums

The physicochemical properties of the purified* Albizia *and* Khaya *gums studied are presented in Table 2.* Khaya *gum gave a higher purification yield, true density, moisture content, and insoluble matter compared to* Albizia *gum.* Albizia *gum also exhibited higher ash values and greater solubility in four of the solvents tested than* Khaya *gum. In addition,* Albizia* gum produced greater swelling capacity and charring temperature than* Khaya *gum. The moisture content and insoluble matter of the two gums were less than 15% and 0.5%, respectively, and hence complied with the official specifications for natural gums [25]. The moisture content affects the flow properties and microbiological stability of gum powders. This is because high moisture content enhances microbial growth and causes some powders to clump and form hard aggregates. The insoluble matter in gums is the result of the mode of gum collection or the foreign materials collected in the gum exudates as they remain on the bark [34].

*Khaya *gum mucilage was more acidic than* Albizia* mucilage and confirms previous findings about the acidity of the two gums [19]. The pH of the gums determines whether they can cause oromucosal irritation when administered as ODFs. Also, the pharmaceutical applications of natural gums such as thickening and suspending agents which are dependent on their viscosity also tend to be pH-dependent. The ash values provide valuable information about the presence of inorganic and other extraneous materials in a natural product and are helpful in aiding the detection of product adulteration. The acid insoluble ash values of <1% indicate that the gums contain insignificant amount of earthly materials due possibly to the proper processing and cleaning of the gum exudates after collection. The gums exhibited high solubility in water at low temperatures and the solubility was enhanced in warm water due to the acquisition of more kinetic energy at elevated temperatures which makes them more mobile to interact with the solvent molecules. On the other hand, the gums were practically insoluble in acetone, ethanol, and chloroform.

The swelling capacity is an important characteristic of ODFs as the dosage form will have to absorb water, increase in size, and disintegrate in order to release the drug for dissolution and subsequent oromucosal absorption [1]. The two gums being hydrocolloids demonstrated excellent swelling capacities. In aqueous media, the gums will absorb water and swell to varying sizes depending on the pH, ionic strength, and the presence of salts in the medium. The true density of a solid is defined as the mass per unit volume exclusive of all voids that are not part of the molecular packing arrangement [26] and is employed in the identification of solid materials. The temperature at which* Albizia* and* Khaya *gums changed appearance (charring temperature) without melting was 258°C and 243°C, respectively. The charring temperature is partly affected by the density and moisture content of the gum. It can be applied in product development to set limits for important parameters like formulation procedures and storage conditions that can affect the appearance of the gum.

Elemental analysis of the two gums showed the presence of the macrominerals calcium, magnesium, sodium, phosphorus, and potassium and the microminerals iron, copper, zinc, and manganese (Table 3).* Albizia* gum was found to contain comparatively higher amounts of iron, copper, manganese, potassium, and zinc than* Khaya *gum. On the other hand,* Khaya *gum had higher levels of calcium and magnesium than* Albizia *gum. The minerals with the highest concentration in* Albizia *and* Khaya *gums were potassium (2.78 g/100 g) and calcium (0.87 g/100 g), respectively. The mineral content affects the properties of the gums in aqueous media such as viscosity and solubility and also determines the nutritional quality of the gums. The macrominerals and microminerals present in the gums are needed for various metabolic activities in the body. The two gums being organic compounds were found to contain high amounts of carbon with* Khaya *gum having relatively higher levels than* Albizia *gum. As organic compounds the gums require proper storage conditions as they are suitable substrates for microbial growth and contamination.

The elemental analysis showed negligible amounts of the toxic metals cadmium, lead, mercury, cyanide, and arsenic in the two gums. These toxic metals have no known function in the body but are very harmful to the proper functioning and metabolic activities of the human body. The near absence of these toxic metals gives a good indication of the possible safety of the gums when used as pharmaceutical excipients.* Albizia *and* Khaya *gums being naturally occurring are readily available and relatively inexpensive and are generally considered to be nontoxic and nonirritant. Characterization of the gums has shown that the two gums also possess the requisite physicochemical properties to be employed as vehicles for the fabrication of diclofenac sodium ODFs.

**Figure 1:**
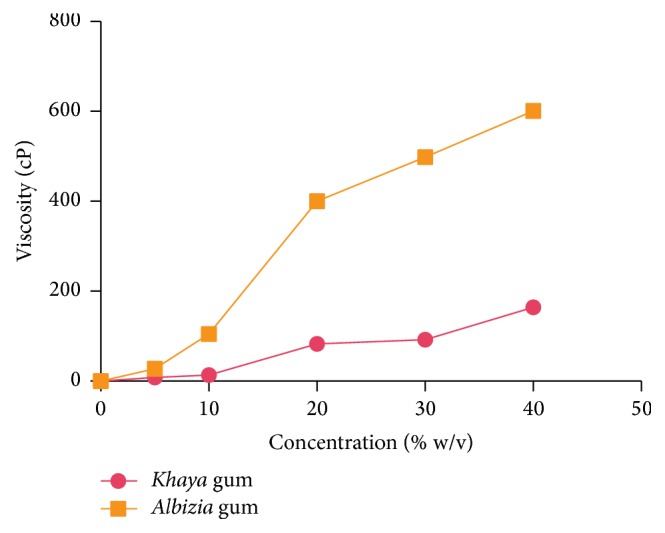
Effect of concentration on the viscosity of* Albizia * and* Khaya *gums.

**Figure 2:**
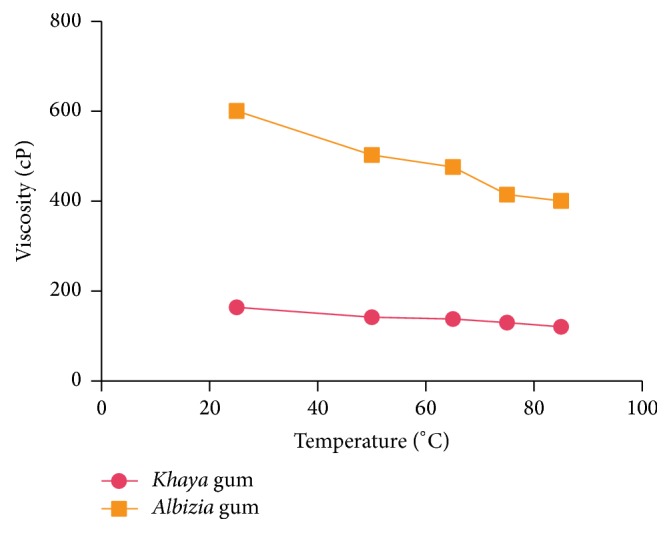
Effect of temperature on the viscosity of* Albizia * and* Khaya *gums.

Figure 1 shows the effect of concentration on the viscosities of the gums. The viscosities of both gums increased with an increase in concentration with* Albizia* gum demonstrating higher viscosities at all concentrations. The effect of temperature on the viscosity of the two gums is shown in Figure 2. The viscosities of both gums decreased with increasing temperature. The higher kinetic energies attained at elevated temperatures possibly facilitate the cleavage of intermolecular bonds between contiguous layers causing a reduction in viscosity of the gum mucilage.

### 3.2. Preparation and Characterization of Diclofenac Sodium ODFs

In the preparation of diclofenac sodium ODFs, HMPC,* Albizia*, and* Khaya *gums were employed as hydrophilic film forming vehicles while glycerol was used as a plasticizer to improve the flexibility of the films. Aspartame and citric acid were used as sweetening and saliva stimulating agents, respectively. Pineapple flavour was meant to impart the needed flavour to the dosage form. Titanium dioxide (1%) was used to mask the unpleasant appearance of the natural gums while Tween 80 was incorporated as a wetting agent. The films were prepared using the solvent casting method which is simple and economical. All the film ingredients used are safe and are commonly used in the pharmaceutical industry. The formulated ODFs were opaque and whitish because of the titanium dioxide used as colouring agent. The films were thin, soft, and flexible (except F7) with smooth and glossy surfaces.

Table 4 presents the physicochemical properties of the films. All pharmaceutical dosage forms are required to exhibit constant dosage and variations in weight within a batch should fall within specified limits [25]. The individual weights of films within the same ODF formulation varied only slightly as shown by the low standard deviations. The average weight of the films increased as the number of polymers used in producing the films was increased, as per the order: F1, F4, F6 (only one polymer) < F2, F3, F5 (two polymers) < F7 (three polymers). Uniformity of thickness is a key physical parameter in the evaluation of ODFs as it has direct correlation with the precision of dose of the films. The films were generally of uniform thickness with the order of thickness of the various formulations as follows: F6 < F5 < F2 < F1, F2 < F4 < F3 < F7.

The pH of the films was determined to understand their possible effect on the mucous membrane of the mouth upon usage of the dosage form. The pH of a product is determined by the drug and the excipients used in the formulation [35]. Generally, acidic and basic oral formulations can cause inflammation of the oral mucosa [36]; hence formulations of neutral pH are preferred. However, the formulations in the current study were acidic due probably to the diclofenac sodium and the excipients, especially* Albizia* and* Khaya *gum and citric acid used. The final pH of the polymeric solution needs to be adjusted to an almost neutral pH before casting into films to prevent any irritation to the oral mucosal lining when administered.

ODFs are required to release the drug in a controlled and reproducible manner and the primary step towards drug release is through disintegration of the films. The average disintegration time for the formulations was 37.16 to 47.45 s. Although no official specifications and guidelines for the disintegration time of ODFs exist [30], they are expected to have short disintegration times. In the current study, formulations F2 and F7 had the lowest and highest disintegration times, respectively. It was observed that combining the polymers in the ODF formulation slightly prolonged the disintegration time due to possible cross-linking of the polymer molecules and/or increased film thickness which makes them less penetrable to water. The limit of content uniformity of ODFs is 85–115% [5, 10]. The average content of diclofenac sodium in the formulations ranged from 97.58 to 100.41%, meaning all the formulations complied with the assay test.

**Figure 3:**
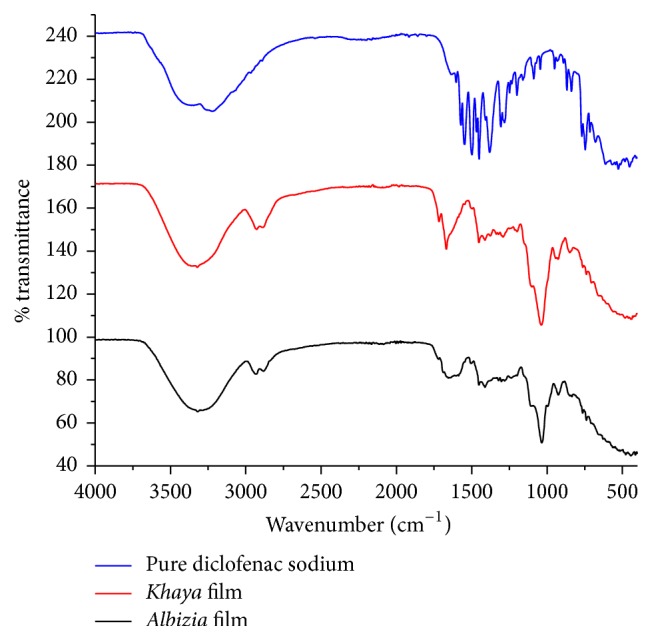
FTIR spectra of pure diclofenac sodium and* Albizia* and* Khaya *gum containing ODFs.

Figure 3 shows the FTIR spectra of pure diclofenac sodium and* Albizia* (F4) and* Khaya *(F6) containing ODFs. The spectrum for pure diclofenac sodium shows a very broad band at 3225.26 cm^−1^ sloping into the aliphatic –CH region around 3000 cm^−1^. This denotes the presence of a carboxylic acid –OH which overlaps, causing the disappearance of the –CH group in the aromatic system. In addition, medium intensity bands at 1603.42 cm^−1^, 1572.03 cm^−1^, and 1547.76 cm^−1^ indicate the presence of aromatic groups and this is confirmed by bending vibrations around 700–900 cm^−1^. All the above principal bands present in the spectrum of diclofenac sodium were present in the ODF formulations produced with* Albizia* and* Khaya *gums. It can therefore be inferred that no interaction occurred between the excipients and diclofenac sodium in the ODFs.

Pharmaceutical ODFs are required to exhibit good mechanical properties in order to maintain their integrity during handling, packaging, and transportation. The mechanical properties can be influenced by the film forming polymer, the technique of film fabrication, and the type and amount of plasticizer used. Plasticizers confer pliability to films and greatly improve their mechanical properties [5]. Tensile strength provides an indication of the mechanical strength and hardness of films [19] and high tensile strength values are desirable.  Table 5 presents the mechanical properties of the diclofenac sodium ODFs. The tensile strength of the formulations ranged from 5.67 to 7.32 MPa. The use of* Khaya *gum alone (F6) produced hard films while* Albizia *gum alone (F4) produced soft films. Addition of HPMC to* Albizia *gum increased the tensile strength of the films (F2) but the inclusion of HPMC to* Khaya *gum rather reduced the hardness of the films (F3).

Elastic modulus describes the stiffness of films [5] and also provides information about how well the film can resist mechanical deformation. Films with low elastic modulus have low rigidity which translates into high elasticity, and vice versa [19]. The range of elastic modulus recorded for the formulations was 3.89 MPa to 5.11 MPa. Generally, a film with high tensile strength and elasticity (low elastic modulus) is preferred because it can withstand stress better and can also resist changes due to mechanical deformation. In the current study, addition of HPMC to both* Albizia * and* Khaya *gums increased their elasticity by decreasing their elastic modulus (F2 and F3). HPMC thus confers flexibility to* Albizia* and* Khaya *films while* Khaya *gum increased the rigidity of* Albizia * films (F5). The highest elastic modulus was observed in the combination of all three polymers (F7) possibly due to the effect of* Khaya *gum as* Albizia*/HPMC combination (F2) showed moderate elastic modulus.

The exertion of stress on a film causes the film to stretch. This effect called strain is expressed as the change in length of a film divided by its initial length before the applied strain [1]. It describes the pliability of films. A low elongation at break signifies a low capacity of the film to resist deformation and hence the film will be easily breakable. A high elongation at break means that the film has a high capacity to withstand mechanical strain. In the current study, the percentage elongation of the formulations ranged from 7.65% to 17.87%. It was observed that HPMC increases the flexibility of both* Albizia *(F2) and* Khaya *(F3) by increasing their respective percentage elongations. A combination of* Albizia* and* Khaya *gum (F5) showed a decrease in flexibility compared to* Albizia* films alone (F4). It can therefore be deduced that* Khaya *gum produces brittle films and its combination with other polymers increases their inelasticity.

Folding endurance expresses the capacity of the film to resist breaking when folded repeatedly along the same plane. High folding endurance values portray considerable mechanical strength of the film. It is directly regulated by the type and amount of plasticizer used in the formulation. The folding endurance of the films was in the order, F6 < F7 < F3 < F4 < F5 < F1 < F2. Folding endurance of 300 is considered satisfactory for oral films [37]; hence the plasticizer concentration could be increased to improve the folding endurance of the films.

**Figure 4:**
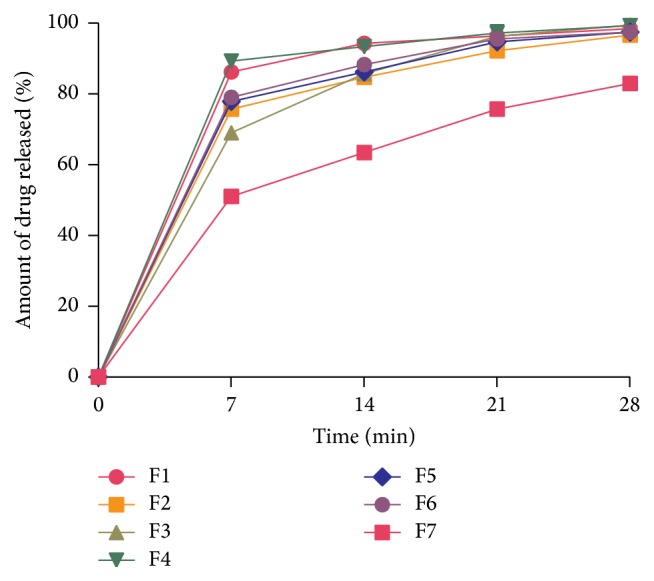
Drug release profiles of diclofenac sodium ODF formulations in phosphate buffer pH 6.8 at 37°C (mean ± SD, *n* = 3).

Figure 4 shows the drug release profiles of the ODF formulations in phosphate buffer pH 6.8 at various time intervals. Although there is no official specification, the dissolving time of ODFs has been defined as the time at which not less than 80% of the film under testing dissolves in aqueous media [38]. Formulations F1 and F4 showed over 80% drug release within the first 7 min and the rest of the formulations (except F7) showed over 80% release in the next 7 min. The release of diclofenac sodium from formulation F7 was slowest due to possible polymer cross-linking between the three polymers used. In general, an increase in the number of polymers used resulted in increased dissolution time of the films.

Table 6 presents the dissolution efficiency and difference and similarity factors of the film formulations. Dissolution efficiency describes how competent a dosage form is in releasing its API for pharmacological effect [39]. The higher the dissolution efficiency, the more capable the dosage form at releasing its deep-seated API. The order of dissolution efficiencies of the formulations was F7 < F3 < F4 < F5 < F2 < F6 < F1. Thus, F1 can be said to be the superior film formulation in terms of drug release. This trend supports the findings from the drug release studies.

The difference factor (*f*1) estimates the percentage difference between two dissolution profiles at every point and is a measure of the relative error between the dissolution profiles of both the test and reference drug. Usually, *f*1 values in the range of 0–15 indicate insignificant difference between the two batches [40]. From the results, all formulations (except F7) had *f*1 values within the desirable range of 0–15. This means that in terms of drug release F2, F3, F4, F5, and F6 were not different from F1, the reference formulation. Formulation F7 however differed from F1 because of its high *f*1 value of 27. In terms of similarity (*f*2), a test formulation is considered similar to the reference if the *f*2 value is in the range of 50–100 [41]. All the formulations (except F7) had *f*2 values within the 50–100 range. Thus, it can be inferred that F2, F3, F4, F5, and F6 are similar to F1 (reference) in terms of their dissolution characteristics and can be used as alternatives.  Table 7 presents the results of one-way ANOVA followed by Dunnett's multiple comparison test on the dissolution profiles of the ODF formulations. No significant difference (*P* > 0.05) was observed in the comparisons of the reference formulation (F1) and formulations F2, F3, F4, F5, and F6. However, there was a significant difference (*P* < 0.001) between F1 and F7.

The kinetic data for the diclofenac sodium ODFs are shown in Table 8. In drug release studies, mathematical models are currently employed to better understand the mechanism of drug release from dosage forms. Advancement in this area has made it possible to predict the release kinetics of drugs based on which mathematical model the dissolution data best fits [33]. The kinetic models employed in the present study were zero-order, first-order, Higuchi, and Hixson-Crowell models. The data from the dissolution studies conducted were fitted into the individual kinetic models to determine their linearity using the coefficient of regressions. In all the film formulations the highest *R* ^2^ values were recorded for the Higuchi model which describes drug release by Fickian diffusion. The Higuchi model is mostly applied to describe drug dissolution from various modified release pharmaceutical dosage forms especially in transdermal systems and matrix tablets with water soluble drugs [42].

## 4. Conclusion

In can be concluded from the study that* Albizia *and* Khaya *gums possess the requisite physicochemical properties for use as film formers for the development of oral dissolvable films. Diclofenac sodium ODFs were successfully formulated using the gums through the solvent casting technique. The ODFs produced generally exhibited satisfactory physicomechanical properties with most of the formulations attaining over 75% drug release in phosphate buffer pH 6.8 within 7 min. The study has demonstrated the potential of* Albizia *and* Khaya *gums as vehicles for the fabrication of diclofenac sodium ODFs and with film properties enhanced through addition of HPMC in the formulation.

## Figures and Tables

**Table 1:** Composition of diclofenac sodium ODF formulations.

Code	^*∗*^Diclofenac sodium (g)	HPMC (g)	*Albizia* (g)	*Khaya* (g)	Gly (g)	Tw (g)	Fla (g)	Asp (g)	Tit (g)	CA (g)	Water (mL)
F1	4	3	—	—	5	1	5	1	1	1	100
F2	4	2	2	—	5	1	5	1	1	1	100
F3	4	2	—	5	5	1	5	1	1	1	100
F4	4	—	5	—	5	1	5	1	1	1	100
F5	4	—	2	5	5	1	5	1	1	1	100
F6	4	—		10	5	1	5	1	1	1	100
F7	4	1	2	5	5	1	5	1	1	1	100

^*∗*^Each 4 cm^2^ film contains ~50 mg diclofenac sodium; Gly = glycerol; Tw = Tween 80; Fla = pineapple flavour; Asp = aspartame; Tit = titanium dioxide; and CA = citric acid.

**Table 2:** Comparative physicochemical properties of purified *Albizia* and *Khaya* gums (mean ± SD, *n* = 3).

Parameter	Type of gum	
	*Albizia*	*Khaya*
Extraction yield (%)	39.38 ± 2.35	67.50 ± 2.98
Moisture content (%)	12.42 ± 2.10	13.92 ± 1.51
Insoluble matter (%)	0.275 ± 0.041	0.282 ± 0.013
pH (1% w/v @ 25°C)	5.00 ± 0.12	3.84 ± 0.09
Total ash (% w/w)	7.853 ± 0.064	5.603 ± 0.023
Water soluble ash (% w/w)	1.303 ± 0.023	1.200 ± 0.017
Acid insoluble ash (% w/w)	0.600 ± 0.017	0.607 ± 0.012
Solubility (%)		
Cold water	1.273 ± 0.064	0.380 ± 0.020
Warm water	1.747 ± 0.083	0.527 ± 0.064
Acetone	0.110 ± 0.044	0.127 ± 0.031
Chloroform	0.070 ± 0.062	0.020 ± 0.020
Ethanol	0.093 ± 0.023	0.087 ± 0.042
Swelling index (%)	611.29 ± 4.07	477.97 ± 8.67
True density (g/mL)	1.363 ± 0.012	1.412 ± 0.073
Temperature of charring (°C)	258.33 ± 2.89	242.67 ± 2.52

**Table 3:** The mineral and toxic metal ion content of *Albizia* and *Khaya* gums (mean ± SD, *n* = 3).

Parameter	Type of gum	
	*Albizia*	*Khaya*
*Mineral ions*		
Iron (mg/100 g)	16.450 ± 0.087	9.640 ± 0.069
Copper (mg/100 g)	3.240 ± 0.069	2.087 ± 0.023
Manganese (mg/100 g)	18.933 ± 0.058	2.637 ± 0.064
Zinc (mg/100 g)	7.883 ± 0.029	6.633 ± 0.058
Calcium (g/100 g)	0.413 ± 0.012	0.870 ± 0.035
Magnesium (g/100 g)	0.633 ± 0.029	0.847 ± 0.046
Potassium (g/100 g)	2.777 ± 0.064	0.193 ± 0.006
Sodium (g/100 g)	0.133 ± 0.012	0.070 ± 0.000
Phosphorus (g/100 g)	0.070 ± 0.000	0.103 ± 0.006
*Other elements*		
Nitrogen (g/100 g)	1.137 ± 0.012	0.560 ± 0.017
Carbon (g/100 g)	45.903 ± 0.023	47.303 ± 0.040
*Toxic metal ions*		
Lead (*μ*g/g)	0.010 ± 0.000	Nil
Mercury (*μ*g/g)	0.005 ± 0.007	Nil
Cadmium (*μ*g/g)	0.015 ± 0.007	0.005 ± 0.007
Arsenic (*μ*g/g)	Nil	0.012 ± 0.002
Cyanide (*μ*g/g)	Nil	Nil

**Table 4:** Physicochemical properties of diclofenac sodium ODF formulations.

Code	^*∗*^Weight (g), *n* = 10	Thickness (mm), *n* = 5	pH, *n* = 5	Disintegration time (s), *n* = 5	Assay (%), *n* = 5
F1	0.186 ± 0.019	0.128 ± 0.013	4.66 ± 0.452	43.19 ± 0.077	98.74 ± 0.448
F2	0.250 ± 0.016	0.128 ± 0.015	5.08 ± 0.181	37.16 ± 0.043	99.46 ± 0.337
F3	0.194 ± 0.023	0.142 ± 0.008	4.82 ± 0.084	41.23 ± 0.070	99.94 ± 0.388
F4	0.208 ± 0.019	0.138 ± 0.023	3.80 ± 0.035	40.32 ± 0.081	100.41 ± 0.212
F5	0.254 ± 0.011	0.126 ± 0.013	3.09 ± 0.026	38.33 ± 0.053	99.68 ± 0.542
F6	0.116 ± 0.011	0.108 ± 0.008	3.62 ± 0.060	43.13 ± 0.058	97.58 ± 0.231
F7	0.298 ± 0.095	0.174 ± 0.011	5.23 ± 0.043	47.45 ± 0.050	98.82 ± 0.734

^*∗*^2 × 2 cm film.

**Table 5:** Mechanical properties of diclofenac sodium ODF formulations (mean ± SD, *n* = 3).

Code	Tensile strength (MPa)	Elastic modulus (MPa)	Elongation at break (%)	Folding endurance
F1	5.95 ± 0.976	4.20 ± 0.786	17.87 ± 0.432	85 ± 1.57
F2	6.32 ± 0.542	3.89 ± 0.321	17.64 ± 0.156	103 ± 2.06
F3	6.51 ± 0.985	4.39 ± 0.465	13.49 ± 0.768	54 ± 1.56
F4	5.67 ± 0.231	4.05 ± 0.654	15.91 ± 0.563	67 ± 1.45
F5	6.14 ± 0.331	4.46 ± 0.943	10.73 ± 0.105	79 ± 2.15
F6	7.32 ± 0.432	4.86 ± 0.543	8.20 ± 0.445	39 ± 0.98
F7	7.19 ± 0.652	5.11 ± 0.213	7.65 ± 0.154	46 ± 1.57

**Table 6:** The difference factor (*f*1), similarity factor (*f*2), and dissolution efficiency (DE) of the diclofenac sodium ODF formulations.

Code	Difference factor (*f*1)	Similarity factor (*f*2)	Dissolution efficiency (DE) [%]
^*∗*^F1	—	—	95.85
F2	7	61	91.88
F3	7	55	89.27
F4	2	89	89.37
F5	5	66	91.84
F6	4	70	93.01
F7	27	33	82.85

^*∗*^Reference sample.

**Table 7:** Data of one-way ANOVA followed by Dunnett's multiple comparison test on drug release profiles of different ODF formulations.

Code	Statistical significance
F1 versus F2	NS
F1 versus F3	NS
F1 versus F4	NS
F1 versus F5	NS
F1 versus F6	NS
F1 versus F7	SD

NS = no significant difference between the two formulations (*P* > 0.05); SD = significant difference between the two formulations (*P* < 0.001).

**Table 8:** Drug release kinetic models of diclofenac sodium ODF formulations.

Code	Zero-order model		First-order model		Higuchi model		Hixson-Crowell model	
	*K*_0_	*R* ^2^	*K*_1_	*R* ^2^	*K*_H_	*R* ^2^	*K*_HC_	*R* ^2^
F1	32.54	0.8685	0.163	0.8580	34.46	0.9250	0.505	0.8615
F2	60.09	0.9834	0.979	0.9736	63.04	0.9989	0.865	0.9651
F3	87.18	0.9097	0.428	0.8860	85.29	0.9575	1.497	0.8942
F4	29.08	0.9793	0.146	0.9755	30.11	0.9938	0.976	0.9770
F5	57.79	0.9464	0.946	0.9400	58.80	0.9722	0.955	0.9601
F6	51.60	0.9144	0.266	0.9059	55.71	0.9554	0.893	0.9089
F7	92.40	0.9853	0.874	0.9704	96.24	0.9941	1.896	0.9753

*K*_0_, *K*_1_, *K*_H_, and *K*_HC_ are kinetic constants for zero-order, first-order, Higuchi, and Hixson-Crowell models, respectively; *R* ^2^ = correlation coefficient.
